# Supplementary material for: Intracellular vesicle entrapment of nanobubble ultrasound contrast agents targeted to PSMA promotes prolonged enhancement and stability in vivo and in vitro
Source: Nanotheranostics. 2022 Feb 14;6(3):270–85. doi: 10.7150/ntno.64735 (PMC8864252; doi:10.7150/ntno.64735)
Supplement: Supplementary file 1 — Supplementary figures. [file ntnov06p0270s1.pdf]

Supplementary Material

Intracellular vesicle entrapment of nanobubble ultrasound contrast agents targeted to PSMA promotes prolonged enhancement and stability i*n vivo* and i*n vitro*

Reshani H. Perera, Eric Abenojar, Pinunta Nittayacharn, Xinning Wang, Gopal Ramamurthy, Pubudu Peiris, James P. Basilion, Ilya Bederman, and Agata A. Exner *

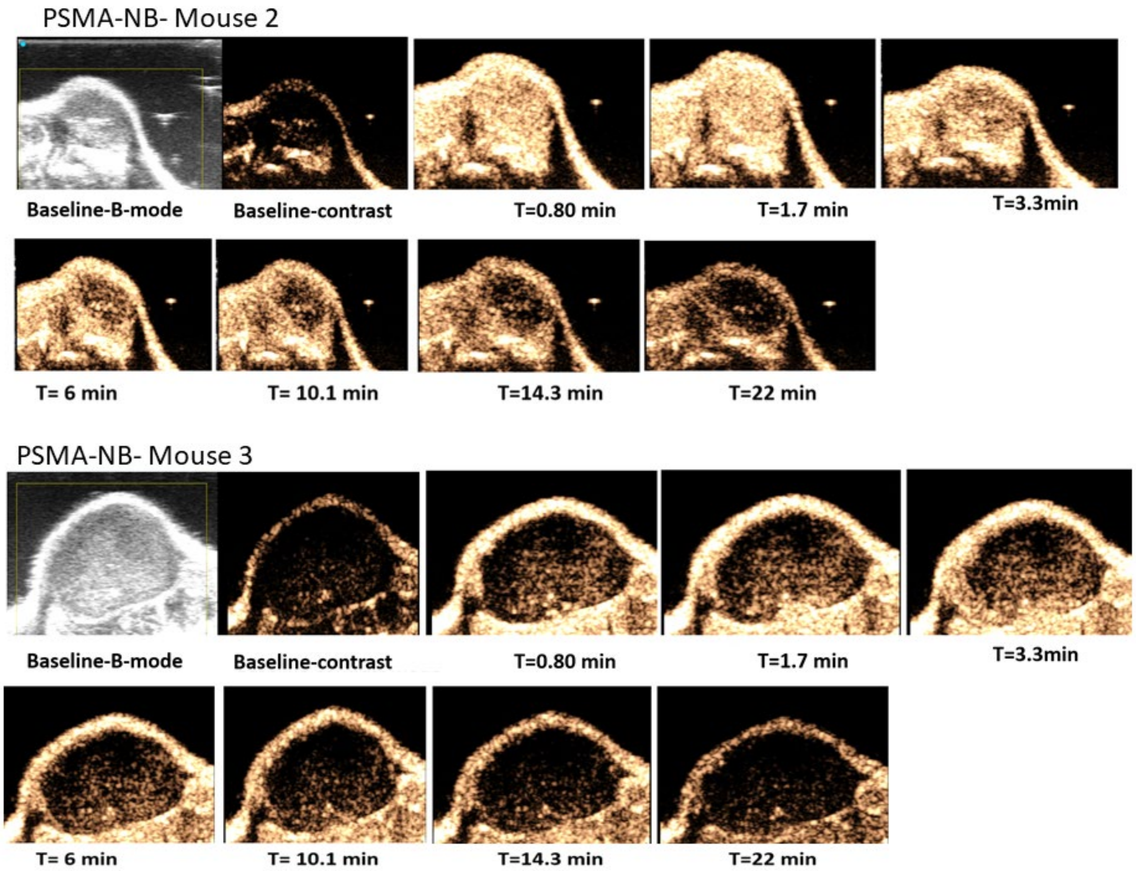

Figure S1. Nonlinear contrast enhanced US images showing **PSMA-NB** distribution in tumors of two additional mice at different times after bubble injection.

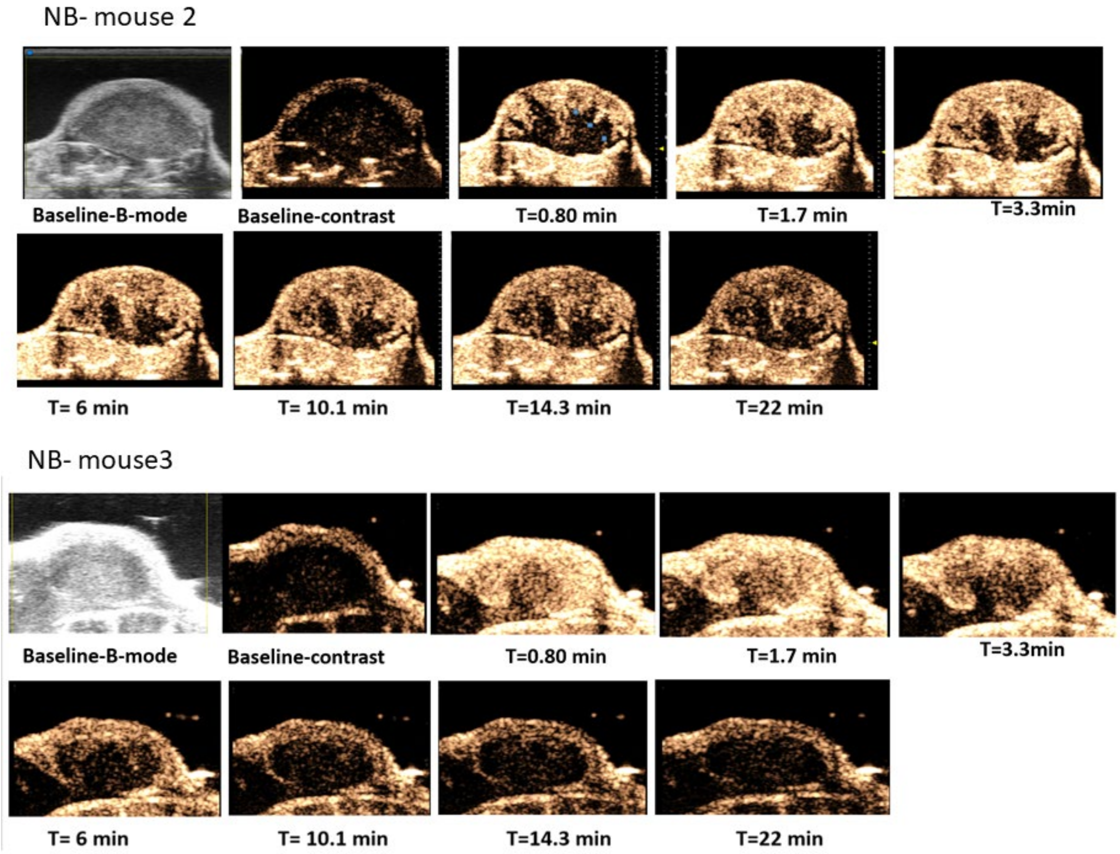

Figure S2. Nonlinear contrast enhanced US images showing **NB** distribution in tumors of two additional mice at different times after bubble injection.

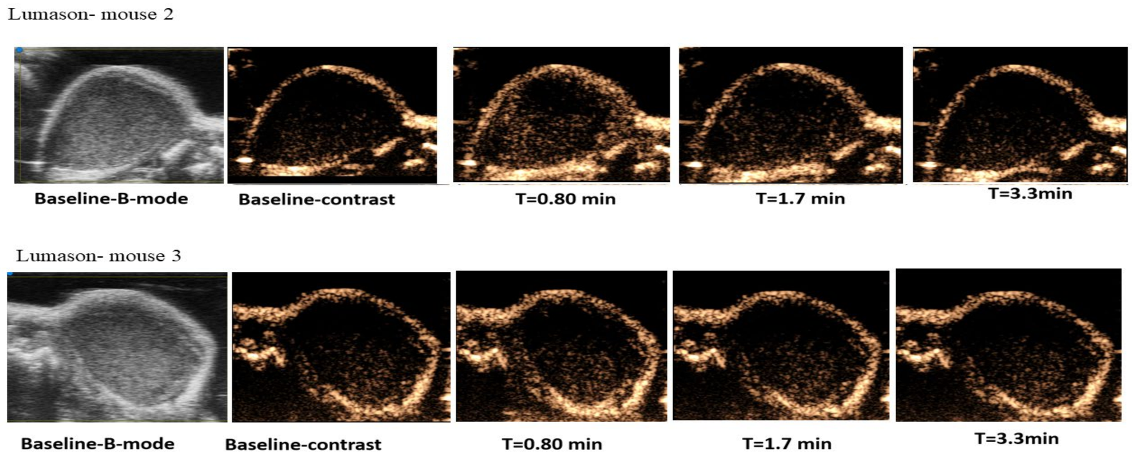

Figure S3. Nonlinear contrast enhanced US images showing **Lumason MB** distribution in tumors of two additional mice at different times after bubble injection.

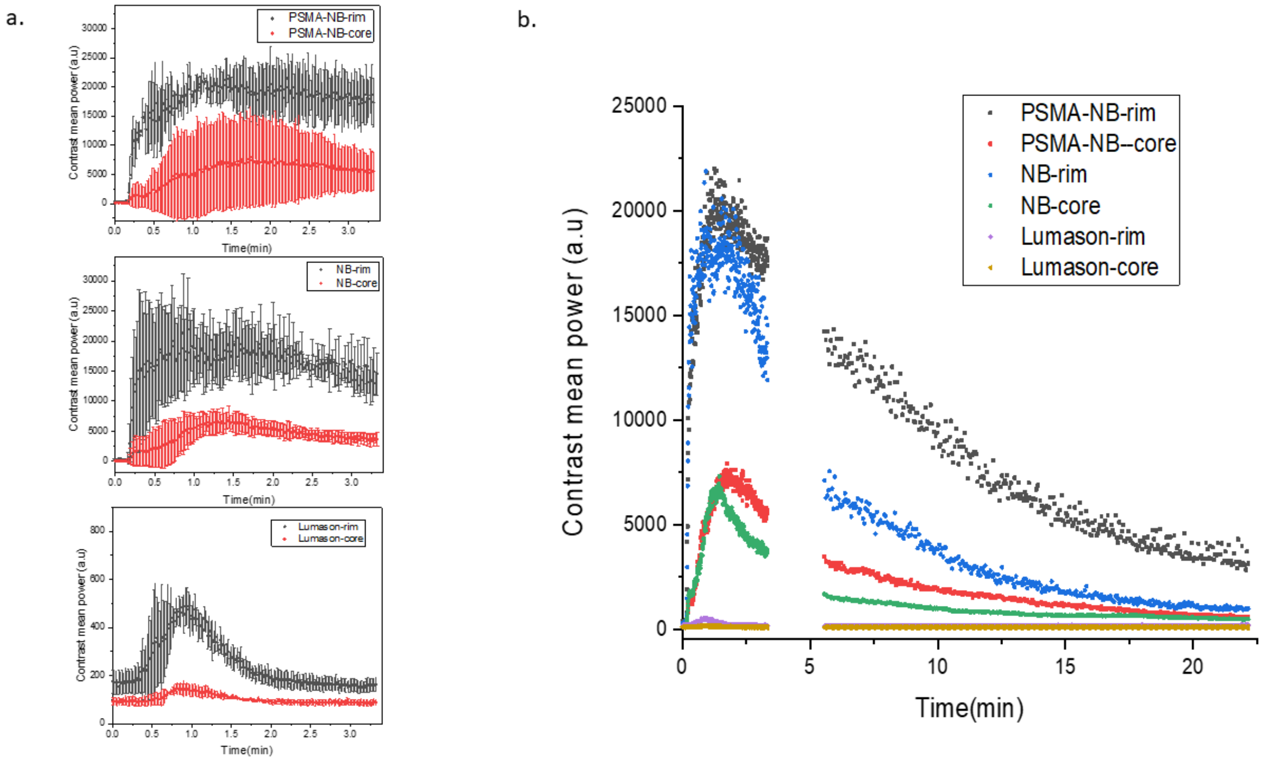

Figure S4. a) Average signal intensity for wash-in phase in tumor rim and core as a function of time for PSMA-NB, plain NB, and Lumason MB. b) Average time-intensity curves for both wash-in and washout phase for PSMA-NB, plain NB, and Lumason MB in tumors (without error bars). Tumors were imaged at 18 MHz, 5 frames/second for 3.33 min and 1 frame/second for 16.67 min.

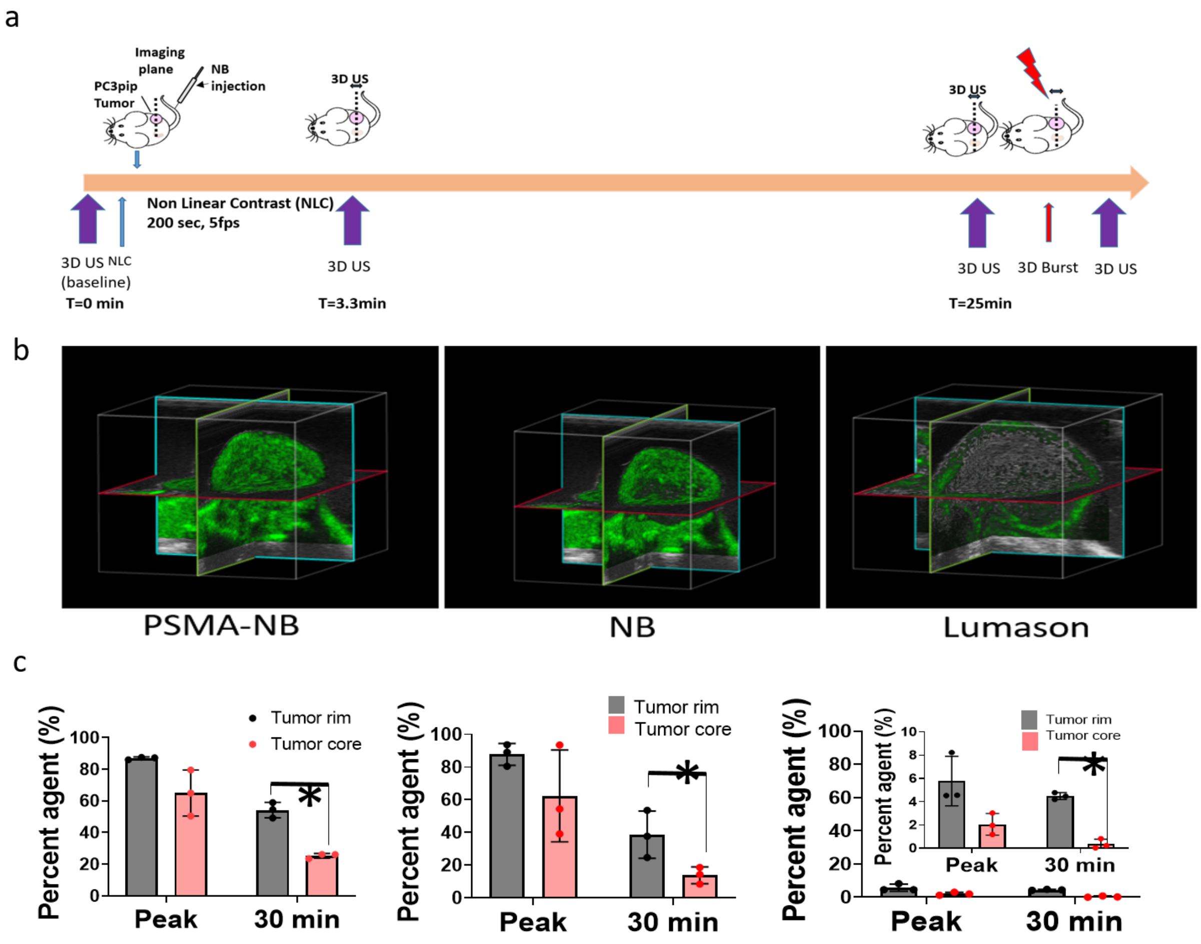

Figure S5. 3D ultrasound scanning to visualize bubble distribution in the entire tumor volume a) Timeline showing the 3D US scanning points. b) Representative 3D US images of the tumor showing PSMA-NB, NB, and Lumason at the peak signal intensity. c) Percent agent coverage per tumor type quantified at peak and the t=30 min after baseline subtraction. n=3, error bars represent mean ± s.d., * $p<0.05$.

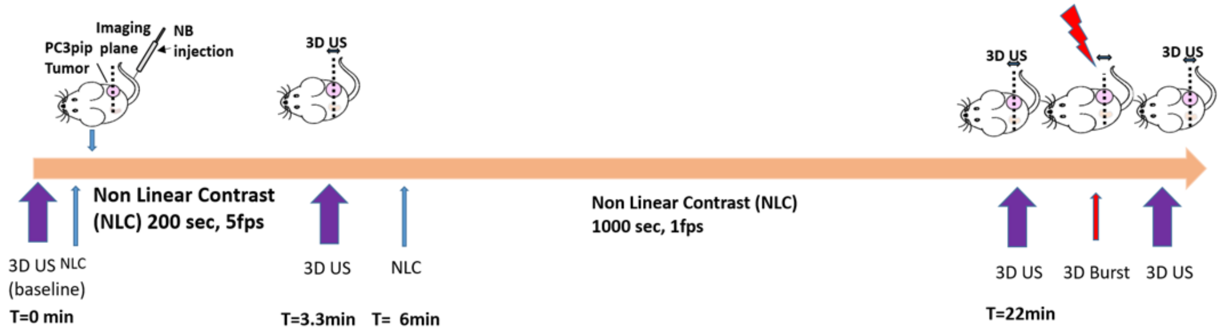

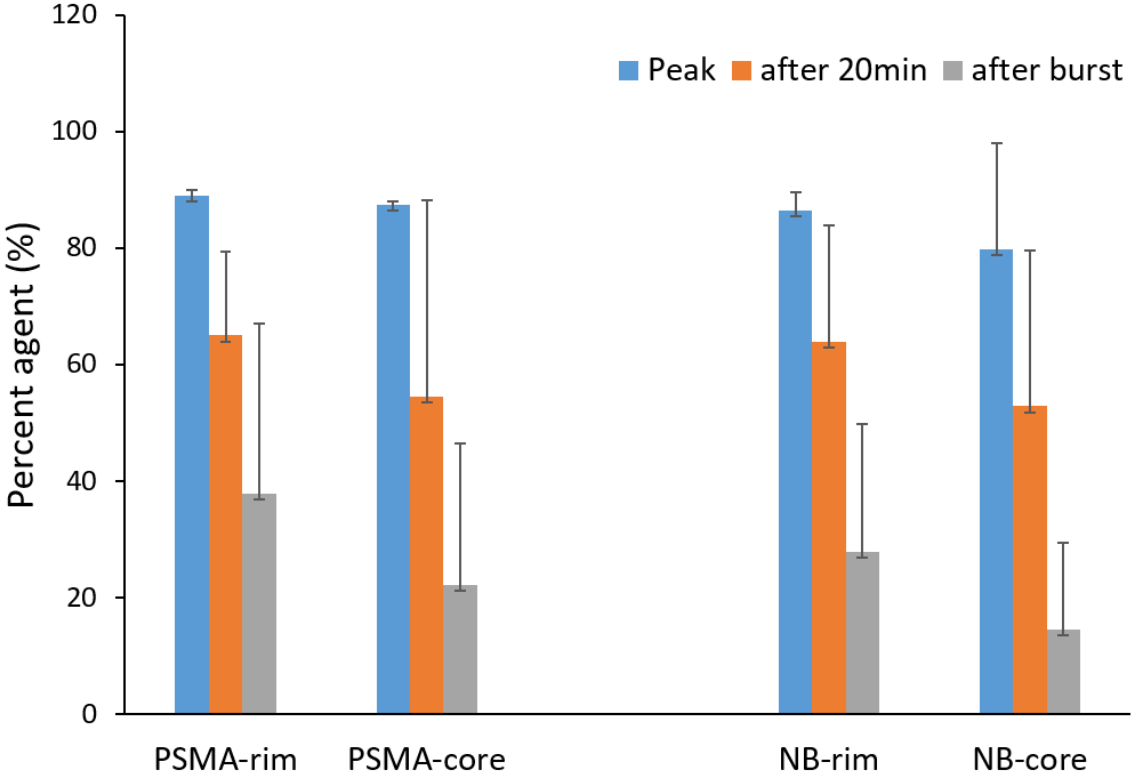

Figure S6. a) Timeline showing the 3D US scanning points. b) Quantification of 3D US signal intensities at peak, at t=22 min, and after burst. The values obtained after subtraction of the baseline value.

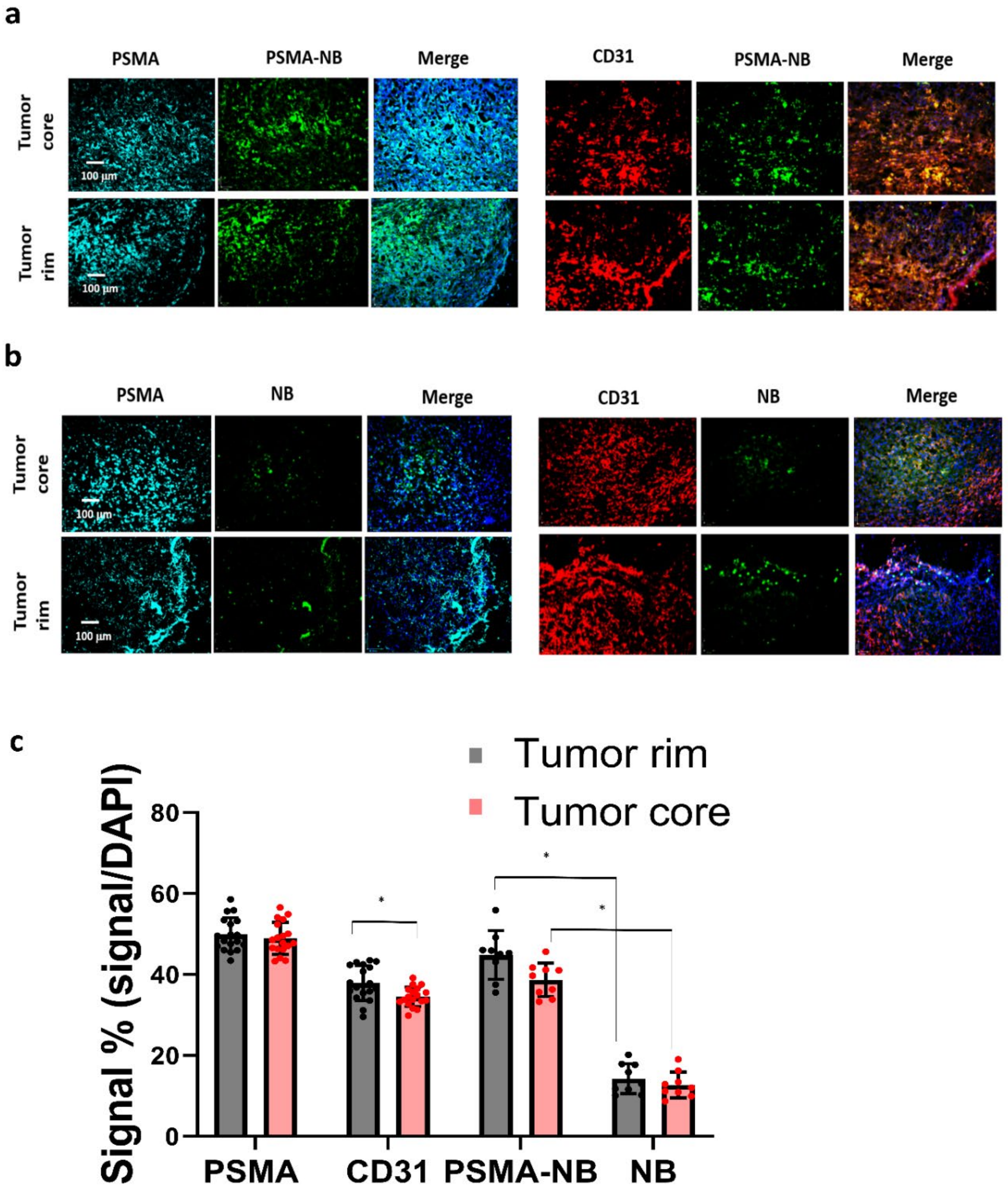

Figure S7. Histology images showing the Cy5.5-PSMA-NB accumulation and extravasation in PC3pip tumor that were excised after cardiac puncture. Representative images (10X magnification) of tumor tissues showing the PSMA expression (cyan), vasculature (CD31 expression, red), and distribution of a) PSMA-NB (green) or b) plain NB (green). c) The signal intensities of bubbles, PSMA and vessel are shown as the percentage of total DAPI (cell) fluorescence in tumor section. Cy5.5-PSMA-NB signal in both tumor rim and core was significantly higher from that of NB. n=3, error bars represent mean ± s.d., * $p<0.001$.

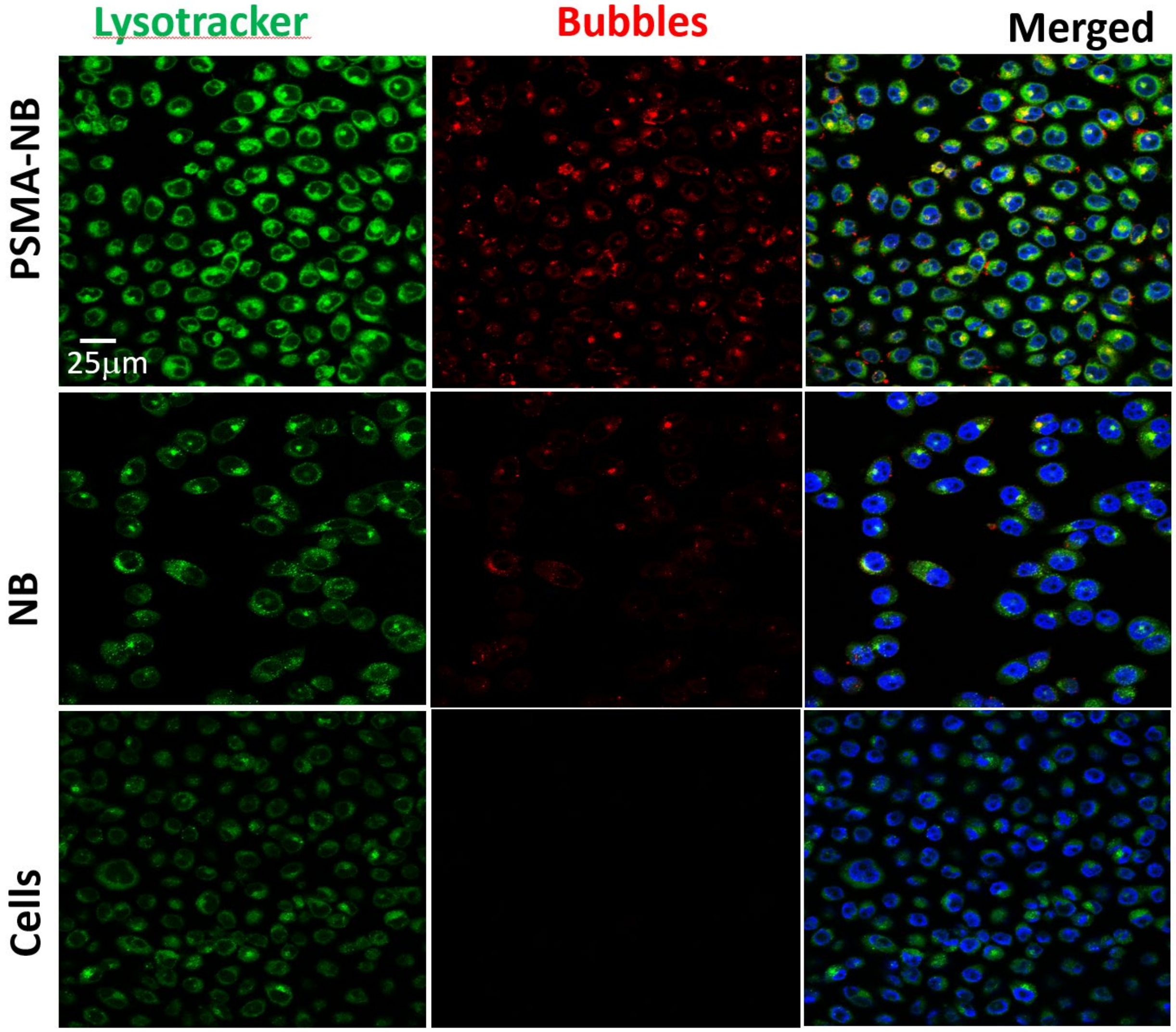

Figure S8. Representative confocal images of PSMA-NB and NB distribution in PC3pip cells after 1h incubation; 40X (blue-nuclei, red-NB, and green-late endosome/lysosomes).

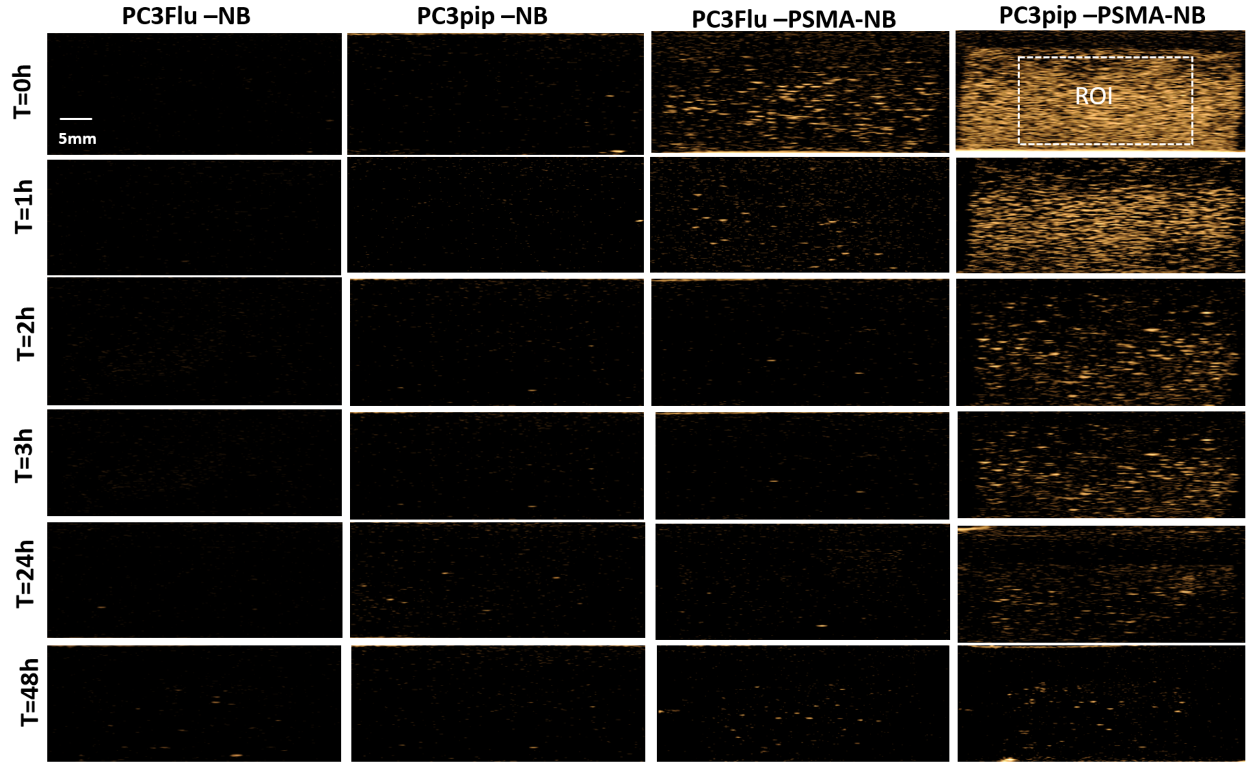

Figure S9. Representative US images of PSMA-NB, NB treated PSMA positive PC3pip cells and PSMA negative PC3flu cells at different times post treatment. PSMA-NB incubated PC3pip shows significantly high acoustic activity at t=0 to t=24 h time points compared to all the other groups. n=3.

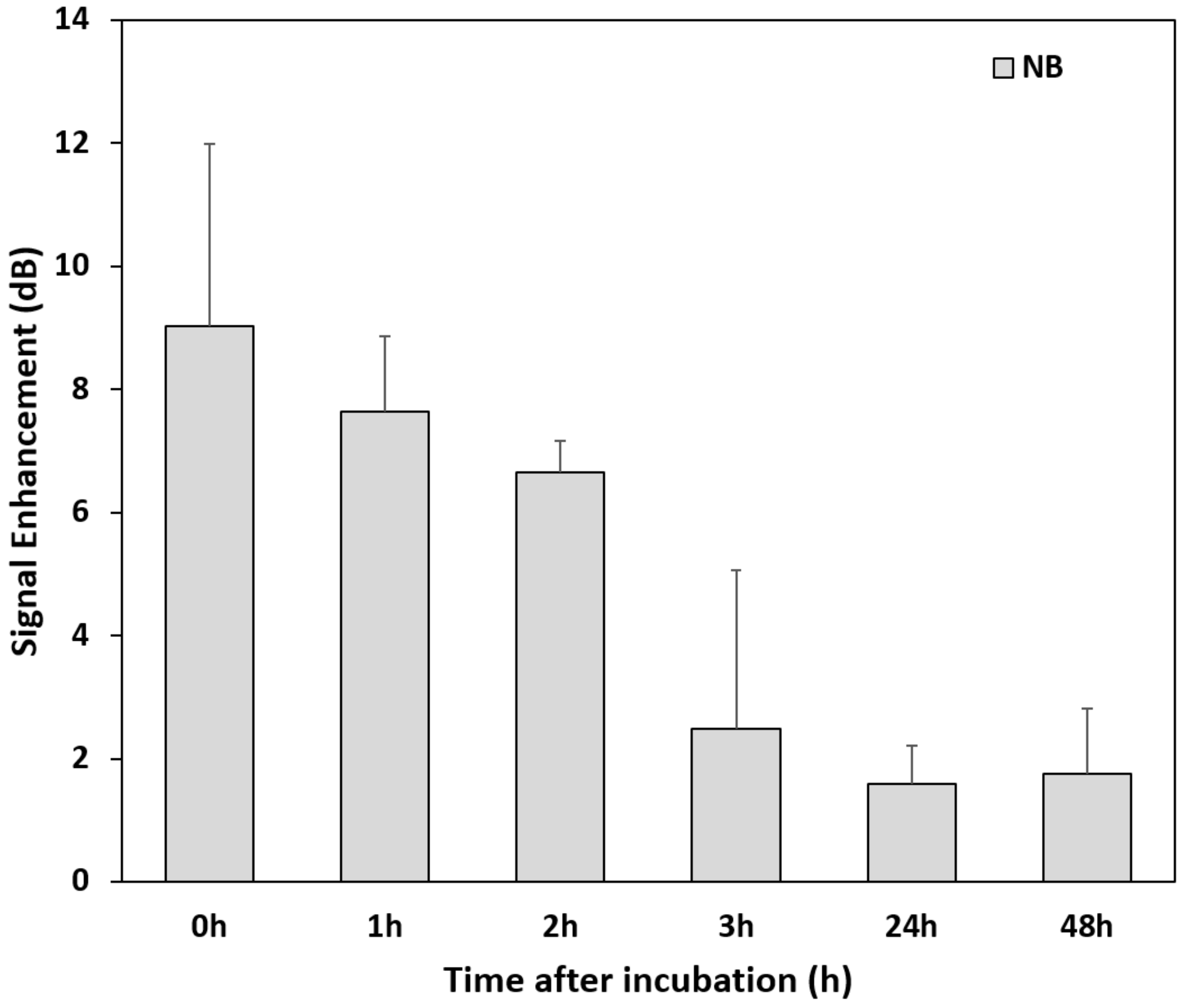

Figure S10. Signal enhancement of PSMA-NB incubated under the same conditions as PSMA-NB internalized cells (at 37 º C and 5 % CO_2) and imaged at different times points.
